# Single Cell Multi-Omics Technology: Methodology and Application

**DOI:** 10.3389/fcell.2018.00028

**Published:** 2018-04-20

**Authors:** Youjin Hu, Qin An, Katherine Sheu, Brandon Trejo, Shuxin Fan, Ying Guo

**Affiliations:** ^1^Zhongshan Ophthalmic Center, State Key Laboratory of Ophthalmology, Sun-Ye-Sat University, Guangzhou, China; ^2^Department of Human Genetics, David Geffen School of Medicine, UCLA, Los Angeles, CA, United States; ^3^The Second Affiliated Hospital, Xiangya School of Medicine, Central South University, Changsha, China

**Keywords:** single cell transcriptome, single cell multi-omics profiling, single cell epigenome, single cell proteome, gene regulation, epigenetics

## Abstract

In the era of precision medicine, multi-omics approaches enable the integration of data from diverse omics platforms, providing multi-faceted insight into the interrelation of these omics layers on disease processes. Single cell sequencing technology can dissect the genotypic and phenotypic heterogeneity of bulk tissue and promises to deepen our understanding of the underlying mechanisms governing both health and disease. Through modification and combination of single cell assays available for transcriptome, genome, epigenome, and proteome profiling, single cell multi-omics approaches have been developed to simultaneously and comprehensively study not only the unique genotypic and phenotypic characteristics of single cells, but also the combined regulatory mechanisms evident only at single cell resolution. In this review, we summarize the state-of-the-art single cell multi-omics methods and discuss their applications, challenges, and future directions.

## Introduction

According to the central dogma, also known as the DNA-RNA-protein axis, DNA provides the code for RNA, which is translated to produce proteins that fulfill biological functions (Crick, 1970). To discover the regulatory mechanisms behind RNA transcription and protein translation, the most straightforward approach is to analyze both DNA and RNA, or both RNA and protein, from the same sample. Despite the complexity of tissues comprised of heterogeneous cell populations, such as cancer, most experimental results to date have been based on analysis of bulk samples, which theoretically read an averaged signal from the population and prevent resolution of cellular variation (Navin et al., 2011; Huang et al., 2015; Gawad et al., 2016). To decipher the mechanism of heterogeneous gene transcriptional regulation, integrated measurement and co-analysis of multiple types of molecules, such as DNA, RNA, and protein, at single cell level is required.

**Figure 1:**
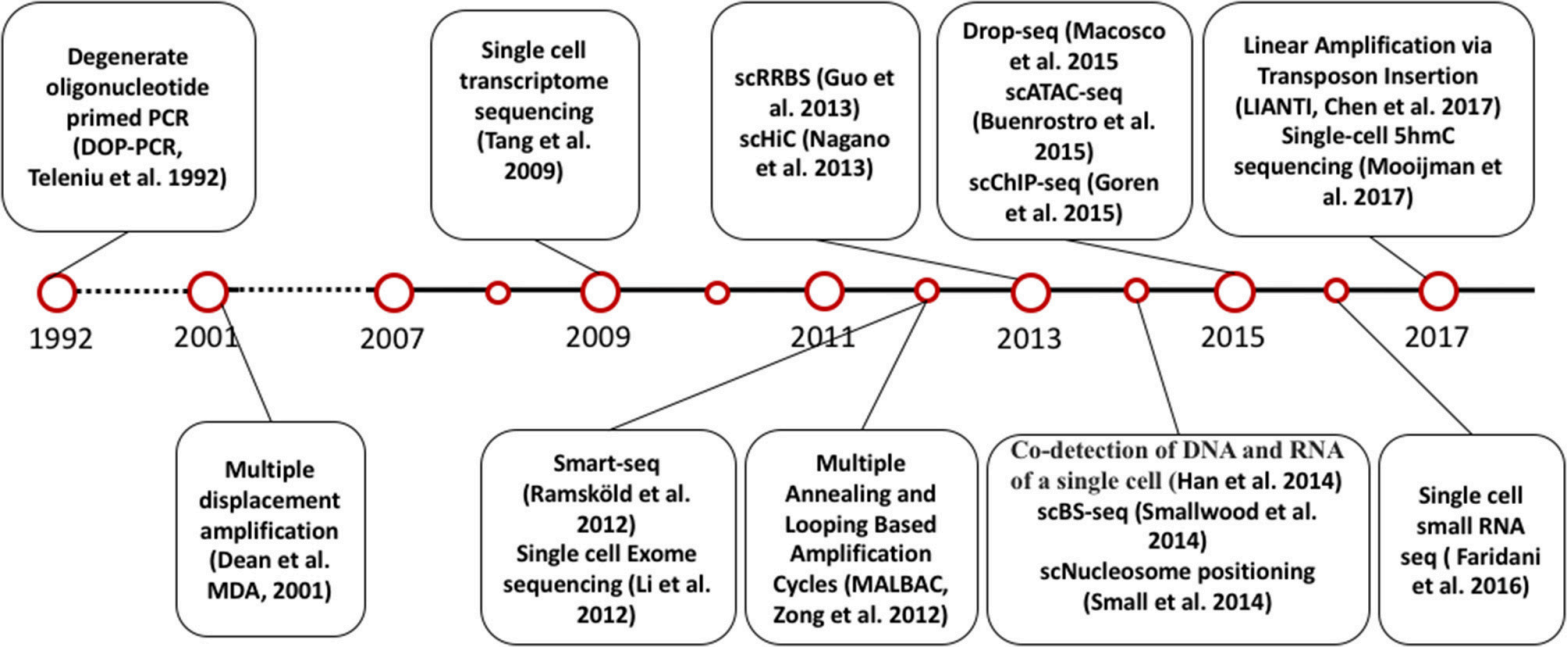
Timeline of single cell sequencing methods milestones.

The invention of PCR methods in 1983 made it possible to analyze the picogram amounts of DNA in single cells, although these initial methods could only amplify small, targeted regions of the genome. However, the development of whole genome amplification (WGA) and whole transcriptome amplification (WTA) methods (Tang et al., 2009; Zong et al., 2012; Huang et al., 2015; Wang and Navin, 2015; Gawad et al., 2016) soon allowed quantitative measurement of DNA and RNA for multiple genes in single cells. At the same time, the development of next generation sequencing technology has enabled genome-wide analysis of DNA and RNA in single cells. Inspired by the very first report of single cell DNA sequencing and single cell RNA sequencing, scientists have developed numerous methods to measure other omics at single cell level, including single cell DNA methylation, single cell chromatin sequencing and single cell proteome analysis [Figure 1, A detailed introduction of single cell sequencing methods has been reviewed elsewhere (Wang and Navin, 2015; Gawad et al., 2016)].

**Figure 2:**
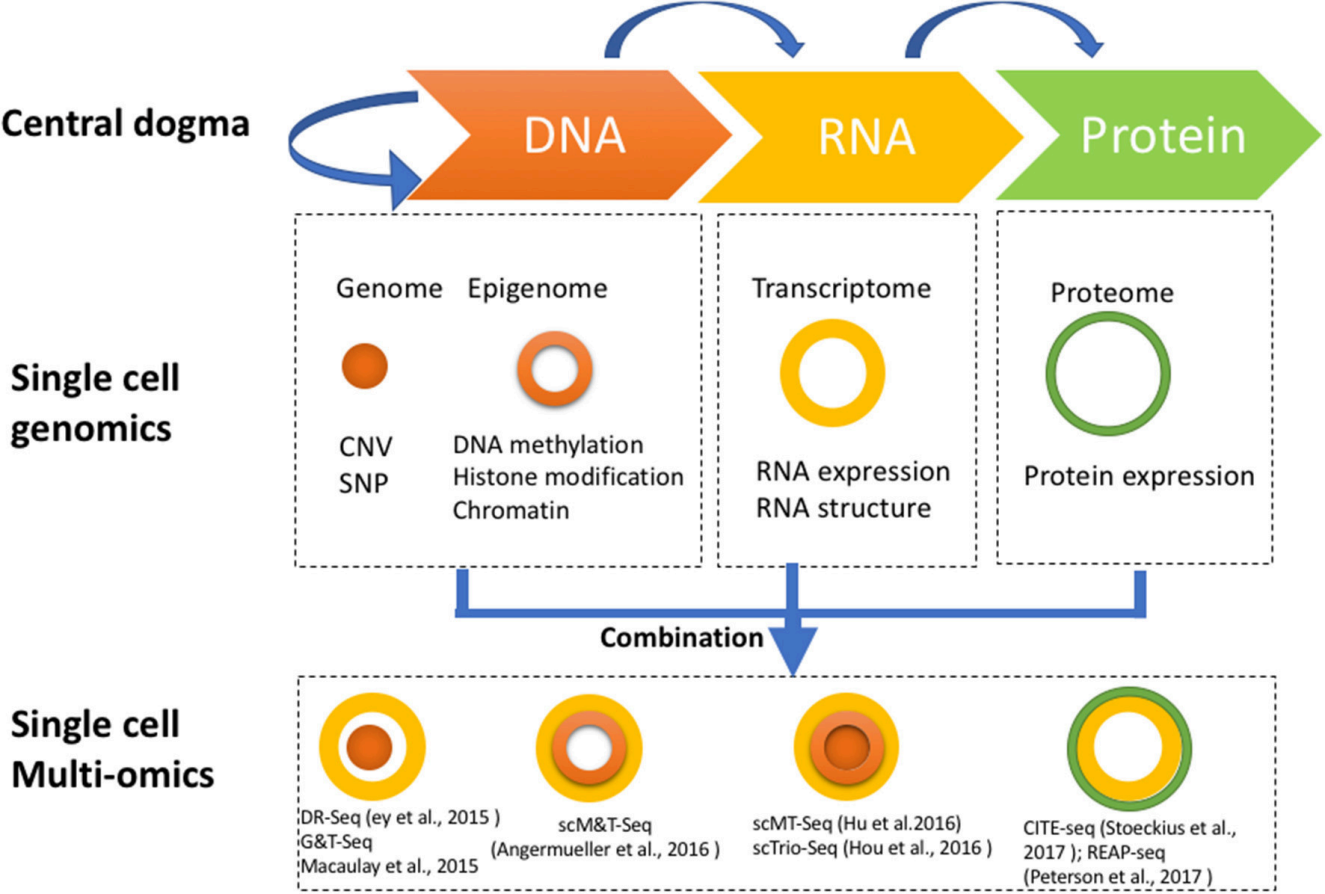
Strategies for multi-omics profiling of single cells. Three major types of molecules relating to biological central dogma **(Top)**. Single cell genomics methods profiling the genome, epigenome, transcriptome, and proteome are shown by different shapes with variable colors **(Middle)**. Single cell multi-omics methods are built by combining different single cell sequencing methods to simultaneously profile multiple types of molecules of a single cell genome wide **(Bottom)**. For example, G&T-seq was built by combining genome (orange) and transcriptome (yellow) to simultaneously detect DNA and RNA of the same cell genome wide.

Single cell genome-wide approaches provide a valuable opportunity to measure different molecules, such as DNA, RNA, protein, and chromatin with ultimate resolution. By isolating multiple types of molecules (DNA, RNA, or protein) from a single cell simultaneously, it is feasible to profile different types of molecules in parallel. For example, genomic DNA can be used to assay the single cell genome, methylome or chromatin accessibility, while RNA from the same cell can be used to profile the transcriptome, and protein the proteome. Utilizing these different single cell omics profiling strategies as building blocks, we can construct a multi-omics profile for the same cell. Here, we summarize current single cell multi-omics approaches, such as scG&T-seq (single cell Genome & Transcriptome sequencing), scMT-seq (single cell Methylome and Transcriptome sequencing), scM&T-seq (single cell Methylome & Transcriptome sequencing), scTrio-seq (single-cell triple omics sequencing), and scCOOL-seq (single cell Chromatin Overall Omic-scale Landscape Sequencing) (MacAulay et al., 2015; Angermueller et al., 2016; Hou et al., 2016; Hu et al., 2016), with each of them measuring a different combination of omics data (Figure 2). We also review the bioinformatics advances that have been necessary to understand the large amounts of multi-dimensional data arising from single cell multi-omics profiling, and we examine the potential for this technology to elucidate numerous biological enigmas.

## Methods for isolating multiple types of molecules from a single cell

Isolating multiple types of molecules from a single cell is the starting point for single cell multi-omics measurement, and generally can be divided into two steps.

The first step is to collect a single cell randomly from a population with heterogeneity. The standard protocol is to get viable, intact cells by mechanical or enzymatic dissociation and then capture single cells from the dissociated cell suspension. Several approaches can be used, including mouth pipetting, serial dilution, robotic micromanipulation, flow-assisted cell sorting (FACS), and microfluidic platforms (Wang and Navin, 2015). Although these collection approaches are borrowed from methods developed for single cell mono-omics sequencing, additional considerations must be taken for multi-omics to ensure that multiple types of molecules can be viably measured in the same cell. The success of this first collection step is critical for preserving an accurate representation of the DNA, RNA, and protein within the cell for downstream measurements. The method used for the initial dissociation of tissues into single cells—mechanical or enzymatic—needs to be selected with consideration for both the nature of the starting material and the types of sequencing to be performed. Clinical samples such as solid tumors are often obtained flash frozen or embedded in paraffin (FFPE), making multi-omics measurements that include cytoplasmic RNA or protein more challenging. However, because this type of freezing process perturbs the cytoplasmic membrane while keeping the nuclear membrane intact, multi-omics measurements that involve the genome, epigenome, and chromatin-associated RNA are still possible after creation of nuclear suspensions (Navin, 2015). For fresh tissues, choice of mechanical or enzymatic dissociation reflects the need for both cell integrity and dissociation quality. Prolonged exposure to common dissociation enzymes such as papain, collagenase, dispase, and neutral protease can result in degradation of RNA and proteins, or generation of cell debris that aberrantly activate cell signaling pathways and cell surface proteins (Autengruber et al., 2012; Volovitz et al., 2016). Mechanical mincing of the starting material through trituration or nanofiltration may also disrupt accurate representation of the proteome or transcriptome in cells that contain long projections such as neurons. These pitfalls in turn can complicate the subsequent computational analyses performed on the data, which often involve identification of correlative relationships among the different layers of multi-omics data obtained. Thus, both tissue-specific and measurement-specific aspects of obtaining multi-omics measurements need to be considered in order to achieve optimized single cell suspensions.

Next, the technique used to select single cells after separation of bulk tissues also has an impact on the feasibility of combinatorial multi-omics measurements. The advantages of techniques such as mouth pipetting and serial dilution include the simplicity and rapidity of moving single cells from the cell suspension to individual reaction chambers. This helps limit the degradation of more volatile molecules such as RNA or protein and may reduce the possibility of non-physiologic changes in chromatin accessibility and chromatin conformation (Wang and Navin, 2015; Svensson et al., 2017). Robotic manipulation, FACS, and microfluidic capture platforms have the advantage of the ability to sort through subpopulations by cell labeling, but require more extensive manipulation of single cells using expensive equipment (Ortega et al., 2017). Of the numerous options, selection of a protocol for isolating single cells for multi-omics data collection will ultimately depend on the molecules that need to be preserved, the type of tissue obtained, and the cost.

The second step is to isolate multiple types of molecules from the same cell, for which there are four main strategies: To isolate DNA and RNA of a single cell, the first strategy is physical separation, including separation of nucleus from cytosol, as genomic DNA is contained in the nucleus and the majority of mRNAs are located in the cytosol. Single cells are treated with a membrane-selective lysis buffer, through which the cell membrane is broken down while the nucleus is kept intact. Then, single nuclei are separated from cytoplasm by micropipetting, centrifugation, or antibody-conjugated magnetic microbeads (Hou et al., 2016; Hu et al., 2016; Han et al., 2018; Table 1). This method has been demonstrated to be highly efficient by several research groups, including our lab. Our data indicates that profiling of cytosolic RNA can resemble the transcriptome of the whole cell. However, this method is low throughput (Hu et al., 2016), as the nucleus-picking procedure is manual and cannot be automated easily. Methods based on centrifugation (Hou et al., 2016) or antibody conjugated magnetic microbeads (Han et al., 2018) can achieve relatively higher throughput in isolating DNA and RNA from single cells.

**Table 1:** Current multi-omics methods.

**Methods (time)**	**Cell type**	**Measurement**	**Approach**	**Single cell isolation**	**Major discovery**	**References**
DR-seq (single cell gDNA and mRNA sequencing) (2015)	Mouse embryonic stem cell line (E14) and breast cancer cell line (SK-BR-3).	Genome, mRNA transcriptome	Amplify gDNA and synthesize cDNA without physically separating the nucleic acids. Product is then split for scWGS (single cell Whole Genome Sequencing) and scRNA-seq.	Mouth pipet	Genome copy number variation could drive transcriptome variability.	Dey et al., 2015
G&T-seq (single cell genome & Transcriptome sequencing) (2016)	HCC38, HCC38-BL and iPSCs carrying trisomy 21.	Genome, mRNA transcriptome	Cell is lysed, genomic DNA and poly(A)+ mRNA is separated by magnetic beads for scWGS and scRNA-seq.	Flow cytometry	Identified transcriptional consequences of chromosomal aneuploidies and inter-chromosomal fusions.	MacAulay et al., 2015
scMT-seq (single cell Methylome and Transcriptome sequencing) (2016)	Mouse dorsal root ganglion neurons.	DNA methylome, mRNA transcriptome	Cell is lysed, cell nucleus containing genomic DNA and cell lysis poly(A)+ mRNA is separated for scRRBS (single cell Reduced Representation Bisulfite Sequencing) and scRNA-seq.	Mouth pipet	Methylation of non-CGI promoters is better anti-correlated with gene transcription, gene body methylation of CGI promoter genes has higher correlation with transcription, potentially reveal allelic specific methylation and allelic expression in single cells.	Hu et al., 2016
scM&T-seq (single cell Methylome& Transcriptome sequencing) (2016)	Mouse embryonic stem cell line (E14), in serum and 2i conditions.	DNA methylome, mRNA transcriptome	Cell is lysed, genomic DNA and poly(A)+ mRNA is separated by magnetic beads for scWGBS (single cell Whole Genome Bisulfite Sequencing) and scRNA-seq (single cell RNA sequencing).	Flow cytometry	Non-CGI promoter methylation and transcription in single cell is negatively correlated; methylation and transcription can be both positively and negatively correlated in distal regulatory regions.	Angermueller et al., 2016
sc-GEM (genotype single cells genotype, gene expression, DNA methylation) (2016)	Human fibroblast, hIPSC, hESC and NSCLC sample.	Genotype single cells while simultaneously interrogating gene expression and DNA methylation at multiple loci	Cell is captured and lysed on C1 Fluidigm chip. RNA is measured using single cell RT-qPCR and methylation is measured using Single Cell Restriction Analysis of Methylation.	Microfluidic device	Tight coupling between the timing of DNA methylation changes and transcription in individual cells; cells have EGFR mutations show a distinct epigenetic signature.	Cheow et al., 2016
scTrio-seq (single-cell triple omics sequencing) (2017)	HepG2 cell line, mESCs and hepatocellular carcinoma tissue sample.	DNA methylome, CNV (copy number variation), mRNA transcriptome	Cell is lysed. DNA and RNA are separated using centrifuge. mRNA is measured using scRNA-seq, methylation is measured using scRRBS. CNV is computationally inferred form scRRBS coverage.	Mouth pipet	Detected subpopulations of cancer cells according to the large-scale CNV, and detected relationships between CNV, methylation and transcription.	Hou et al., 2016
Simultaneous multiplexed measurement of RNA and proteins in single cells (2016)	Cell culture from glioblastoma multiforme patient sample.	Protein and multiple mRNA	Cells are sorted and lysed. Protein is detected by homogeneous affinity-based proximity extension assay (PEA), and RNA is measured by microfluidic qPCR.	Cell sorting and microfluidic devices	RNA and protein data provide complementary information in defining cell states, and significant heterogeneity in cell culture derived from a glioblastoma multiform patient.	Darmanis et al., 2016
scCOOL-seq (single cell Chromatin Overall Omic-scale Landscape Sequencing) (2017)	Mouse preimplantation embryos at different developmental stages.	Chromatin state, DNA methylation, and CNV	Cell is lysed. Chromatin is treated by GpC methyltransferase, and treated DNA is sequenced by scWGBS. *In vivo* methylation is detected as CpG methylation, and chromatin accessibility is computationally inferred by GpC methylation level.	Mouth pipet	DNA methylation is different between paternal and maternal alleles, but their chromatin accessibility states are similar.	Guo et al., 2017
CITE-seq (cellular indexing of transcriptomes and epitopes by sequencing) (2017)	Cord blood mononuclear cells.	Protein and mRNA transcriptome	mRNA is sequenced using 10X genomics platform. Protein is detected by oligo-labeled antibody, which can be read out during sequencing.	Compatible with 10X genomics, adaptable to other platforms	Multimodal data enable to reveal phenotypes that could not be discovered by using scRNA-seq alone.	Stoeckius et al., 2017
REAP-seq (RNA expression and protein sequencing assay)	human lymphocytes	Protein and mRNA transcriptome	mRNA is sequenced using 10X genomics platform. Protein is detected by oligo-labeled antibody, which can be read out during sequencing.	Flow cytometry	assess the costimulatory effects of a CD27 agonist on human CD8+ lymphocytes and to identify and characterize an unknown cell type	Peterson et al., 2017
scNMT-seq (single-cell nucleosome, methylation and transcription sequencing) (2018)	Mouse embryonic stem cells	Nucleosome status, DNA methylation and mRNA transcription	Similar with scM&T methods, DNA and mRNA were isolated. DNA was cut with GpC methyltransferase M.CviPI before bisulfite treatment.	FACS	Novel links between all three molecular layers and revealing dynamic coupling between epigenomic layers during differentiation	Clark et al., 2018
SIDR-seq simultaneous isolation of genomic DNA and total RNA (SIDR) and sequencing. (2018)	Human lung cancer and breast cancer cells, MCF7, HCC827, and SKBR3 cell lines.	Genome, mRNA transcriptome	Nucleus and cytosol of a single cell were separated by antibody-conjugated magnetic microbeads. mRNA is measured using smart-seq2, gDNA is measured using ingle-cell whole-genome amplification (Repli-g single cell kit)	Manually diluted to 48-well	copy-number variations positively correlated with the corresponding gene expression levels	Han et al., 2018

The second strategy uses oligo-dT primer coated magnetic beads to bind and separate polyadenylated mRNA from DNA (MacAulay et al., 2015; Angermueller et al., 2016). Genome wide sequencing of single cell DNA and RNA purified by this method indicated that breadth of genome coverage and number of genes were not affected by the process of separation, indicating high efficiency in the recovery of DNA and RNA. Since this strategy is adaptable to liquid-handling robots or automated work stations, higher throughput can be achieved. However, coverage of isolated DNA was less evenly distributed across the genome compared to that of the whole single cell sequencing, which may result in less accuracy for copy number analysis of certain genomic regions at a suboptimized sequencing depth.

Besides direct physical isolation of DNA and RNA at the beginning, the third strategy is to preamplify DNA and RNA simultaneously, followed by separation into two parts (Dey et al., 2015). Whole transcriptome sequencing of preamplified RNA of one part showed a similar number of genes covered compared to that of whole single cells. However, as the amplified DNA does not retain methylation states, this method is not suitable for methylome analysis.

The fourth strategy is to split the material of a single cell into two parts directly. For example, a recent report used the splitting strategy to split a single cell into two parts and simultaneously analyze the RNA and protein of the same cell (Darmanis et al., 2016). This splitting strategy is not an ideal method to isolate substrates such as DNA because some material will inevitably be lost due to the uneven split. However, for RNA and protein molecules with high copy number in the single cells, this method is feasible as long as the split is even between the two parts.

## Integration of genome and transcriptome

The first single cell transcriptome analysis was reported in 2009 (Tang et al., 2009), and many additional single cell RNA sequencing methods have been developed since, such as Quartz-seq (Sasagawa et al., 2013), smart-seq (Switching mechanism at 5′ end of the RNA transcript) (Goetz and Trimarchi, 2012; Picelli et al., 2014), Cel-seq (Cell expression by linear amplification and sequencing) (Hashimshony et al., 2012) etc., which were developed using different strategies for different purposes. For example, Quartz-seq detects the 3′ end of transcripts, while Smart-seq detects full length transcripts. Cel-seq barcodes and pools samples before linearly amplifying mRNA to multiplex single cell samples. In parallel, due to the development of single-cell whole-genome amplification (WGA) methods, single cell genome sequencing technologies have also been established. At present, four major WGA methods have been reported: DOP (degenerate oligonucleotide-primed polymerase chain reaction) (Telenius et al., 1992), MDA (Multiple Displacement Amplification) (Dean et al., 2001), MALBAC (Multiple Annealing and Looping Based Amplification Cycles) (Zong et al., 2012) and PicoPLEX (Rubicon Genomics PicoPLEX Kit). In 2013, Han et al. first reported a co-detection of DNA and RNA from the same single cell (Han et al., 2014), which was achieved by physical isolation of cytoplasm (containing cytoplasm RNAs) from nucleus (containing the intact genome) from the same single cells, followed by separate amplification of the transcriptome and genome, and further by respective sequencing of both. Although the initial report showed only the data of the whole transcriptome but not the whole genome, instead of Sanger sequencing of a selected set of genomic sequences, it paved a way to establish multi-omic profiling methods. Later, experimental protocols that simultaneously sequenced the genome and transcriptome were developed by elegantly integrating existing single cell sequencing methods, namely DR-seq (gDNA and mRNA sequencing) (Dey et al., 2015) and G&T-seq (Genome & Transcriptome sequencing) (MacAulay et al., 2015). In DR-seq, a cell is lysed completely, releasing its DNA and RNA into the same reaction system. Genomic DNA and cDNA initially being amplified at the same time is split into two halves: one for RNA-seq using the CEL-seq protocol, and the other half for genome sequencing using MALBAC (Dey et al., 2015). Different from DR-seq, G&T-seq separated poly-A tailed mRNAs from DNA by using oligo-dT-coated magnetic beads. Separated mRNA and DNA were then sequenced using SMART-seq2 and various WGA protocols (MDA or PicoPLEX), respectively (MacAulay et al., 2015). Most recently, Han et al. reported a novel method for simultaneous isolation of genomic DNA and total RNA (SIDR) from single cells by using hypotonic lysis to preserve nuclear lamina integrity and subsequently capturing the cell lysate using antibody-conjugated magnetic microbeads. They found that copy-number variations positively correlated with the corresponding gene expression levels (Han et al., 2018). In summary, using DR-seq, G&T-seq and SIDR, researchers were able to directly determine the correlation between large-scale copy number variation and transcription levels in the CNV regions.

As discussed previously by MacAulay et al. (2017), a substantial advantage of direct measurement of multiple molecular types from the same single cell over separate measurement of each type of molecule from different cells is that genotype-phenotype correlation can be determined unambiguously. First, the genomic variation can be directly linked to the transcriptional variation without being confounded by cell heterogeneity, enabling the dissection of potential molecular mechanisms underlying variable phenotypes among single cells. Second, coupled with lineage record technology, simultaneous sequencing of the genome and transcriptome can be used for reconstruction of lineage trees. Genomic profiling of single cells can divulge the lineage relationship among single cells, based on inherited mutations. The transcriptome profiling of the same single cells can in parallel provide information about the cell's phenotype and function. One intriguing application of this method is to dissect the mechanism of heterogeneity of tumor cells to inform our knowledge of tumor formation and potential therapeutic targets (Shapiro et al., 2013). Third, simultaneous sequencing of DNA and RNA of the same cell can detect DNA mutations with higher accuracy, as the mutations found in DNA or RNA can be verified by each other. This strategy can be very helpful in situations where highly accurate mutation calling from a single cell is required, such as genetic diagnosis screening during *in vitro* fertilization, when only 1–2 single blastomeres are available (Vermeesch et al., 2016). Of note, post-transcriptional modification such as RNA editing (Tan et al., 2017) which may affect the concordance of variations in both DNA and RNA, should be taken into consideration to precisely call the mutations.

## Integration of epigenome with transcriptome

Based on the development of technologies for single cell epigenome and transcriptome profiling, the methods for the integrated analysis of the epigenome and transcriptome were developed (Angermueller et al., 2016; Hou et al., 2016; Hu et al., 2016). DNA methylation has been demonstrated to have key regulatory functions on gene expression in many biological process, so the relationship between the DNA methylome and transcriptome from the same single cell is of great interest. Two major methods for single cell methylome analysis are single cell reduced representative bisulfite sequencing (scRRBS) (Guo et al., 2013) and single cell whole genome bisulfite sequencing (scWGBS) (Smallwood et al., 2014). The first reported combined DNA methylome and transcriptome profiling method is scM&T-seq (single cell methylome and transcriptome sequencing), which is developed using the procedure of G&T-seq to isolate DNA and RNA from the same single cell. The protocols for mRNA capture, amplification and sequencing are the same as those in G&T-seq. In parallel, the genomic DNA is subjected to bisulfite treatment and sequencing, allowing the simultaneous profiling of the DNA methylome and RNA transcriptome from the same single cell (Angermueller et al., 2016). Subsequently, scMT-seq (Hu et al., 2016) and scTrio-seq (Hou et al., 2016) were reported using a different strategy to isolate DNA and RNA from a single cell, in which cell membrane but not nucleus was selectively lysed to release RNA, and then intact nucleus was physically separated from the cell lysate (Hou et al., 2016; Hu et al., 2016; Guo et al., 2017). In the scMT-seq method, the single cell nucleus is collected by micropipette and subjected to scRRBS, and mRNA in the lysate is amplified by a modified Smart-seq2 protocol. In the scTrio-seq, the nucleus and cytosol are separated by centrifugation, and genomic DNA contained in the nucleus is sequenced by scRRBS while mRNA is amplified by the scRNA-seq protocol reported by Tang et al. (2009).

The simultaneous profiling of methylome and transcriptome of a single cell provides a unique opportunity to directly measure DNA methylation and gene transcription within the same single cell, and to study the correlation of DNA methylation differences with gene transcription variance across single cells. For example, scM&T-seq investigated the relationship between the transcriptome and DNA methylome, and found that low methylated regions (LMR) showed high variance in methylation level, which is consistent with their role as distal regulatory elements that control gene expression (Angermueller et al., 2016). Our results using scMT-seq found that variable CpG sites were significantly enriched at non-CGI (non-CpG island) promoters but depleted at CGI (CpG island) promoters, suggesting that non-CGI promoters could be the major region contributing to methylome heterogeneity among dorsal root ganglion single cells. We also found that transcription level was positively correlated with genebody methylation, but negatively correlated with promoter methylation. In addition, by integrating the genomic SNP information, we found a correlation between allelic gene body methylation and allelic expression at single cell level. Thus, scMT-seq allows us to profile genome, DNA methylome and transcriptome in parallel within a single cell (Hu et al., 2016). Similarly, scTrio-seq enables profiling of DNA methylome, genome (CNV) and transcriptome at the same time, in which the copy number variation is computationally inferred from the scRRBS (Hou et al., 2016). Most recently, Guo et al. from the same group reported another single cell multi-omics sequencing method called single-cell COOL-seq that can profile DNA methylation and chromatin state/nucleosome positioning, copy number variation and ploidy simultaneously from the same cell (Guo et al., 2017). Although they did not incorporate the RNA sequencing in this protocol (which is theoretically possible), this method provided new insights into the comprehensive study of genome-wide gene regulation at single cell level. Most recently, Clark et al. reported the scNMT-seq (single-cell nucleosome, methylation, and transcription sequencing), which can simultaneously profile single cell nucleosome, DNA methylation and transcription. By profiling the mouse embryonic stem cell, they found novel links between all three molecular layers and revealed dynamic coupling between epigenomic layers during differentiation (Clark et al., 2018).

## Parallel profiling of RNA and protein

RNA and protein have distinctive biochemical properties. Compared to genomic sequencing methods, the throughput in terms of the number of proteins that can be detected by the single cell proteome profiling is limited. Until now, a few single cell proteomic methods have been developed based on different strategies, including fluorescence-activated cell sorting (FACS), western blot, metal-tagged antibodies followed by mass cytometry, and oligonucleotide labeled antibodies. Although the multiplexing of these approaches were still limited to tens of proteins for a single cell, they still demonstrated the feasibility of detection of protein and RNA expression, paving a way to discover the dynamics of RNA and protein within the same cell. Darmanis et al. developed a method based on homogeneous affinity-based proximity extension assay that converts protein abundance into tag-oligo levels (Darmanis et al., 2016), and both transcript level and protein level were quantified by qPCR. This method has succeeded in capturing parallel profiles of protein and RNA for up to 96 genes (Darmanis et al., 2016). Another approach to simultaneously detect the RNA and protein of the same cell is PLAYR (proximity ligation assay for RNA). Briefly, the RNA transcripts are bound by and ligated to isotope labeled probes. Transcript levels are converted into isotope label levels that can be easily measured together with elemental isotope-labeled protein using mass cytometry (Frei et al., 2016). With this method, simultaneous quantification of more than 40 different mRNAs and proteins can be achieved, although improvement is required to achieve genome-wide measurement with higher throughput. Most recently, two methods named REAP-seq and CITE-seq with higher throughput have been reported, in which oligonucleotide-labeled antibodies are used to integrate cellular protein and transcriptome measurements into an efficient, single-cell readout (Peterson et al., 2017; Stoeckius et al., 2017). Quantified proteins with 82 barcoded antibodies and more than 20,000 genes can be detected in a single workflow.

## Strategies for bioinformatics analysis of single cell sequencing data

Single cell sequencing technologies for genome wide profiling of DNA and RNA, as well as the subsequent integrative computational analysis methods, are central to the interpretation of single cell multi-omics data. The prelude to this type of analysis hinges first on the development of bioinformatics approaches for single cell single-omics sequencing data for various individual types of molecular measurements. Because technical characteristics of various single cell sequencing protocols are different, the bioinformatics methods involved must also be customized to correctly analyze each data type. The need to address the specific characteristics of different single cell sequencing approaches has inspired many computational methods that allow us to better analyze sequencing datasets involving multiple layers.

### Single cell genome sequencing

Two major purposes of single-cell genome sequencing are identifying copy number variation and identifying point mutations/SNPs. Both these questions have been addressed in bulk WGS, and the methods developed for bulk WGS data have provided guidance for single cell WGS analysis.

Copy number variation can be robustly identified using Hidden Markov Model (HMM) or Circular Binary Segmentation (CBS), and these methods have proved effective for scWGS data (Knouse et al., 2016). Although these two methods perform similarly in many situations, user-defined parameter adjustments within the algorithms can affect the sensitivity and specificity of copy number calls. For example, comparison of these two methods on scWGS data with a range of parameters indicated that CBS was more sensitive in calling copy number losses, while HMM was more sensitive in calling gains (Knouse et al., 2016). In the context of single cell CNV analysis, one strategy to reconcile the two approaches has been to take the overlap of CNVs identified by CBS and HMM to increase confidence (Knouse et al., 2016). Considerations in choosing between the methods involve the biological properties of the samples, such as the expected sizes of the CNVs, which could range from whole-arm changes seen in aneuploid tumors to dinucleotide changes observed in inherited polymorphisms or in microsatellite instability. CBS is more flexible than HMM in that the algorithm recursively searches for segmentation points in an unsupervised approach, while HMM depends on the assumption that segmentation points follow a homogenous Poisson process, which is not always the case and may therefore compromise flexibility (Wineinger et al., 2008).

Many tools have been developed for detecting variations in bulk WGS data (Depristo et al., 2011; Koboldt et al., 2012), and these methods, in principle, should perform well in scWGS data. However, scWGS data suffers from high allele coverage bias and high PCR amplification error, which could impair the performance of variant calling methods if not corrected. Recently, with increased understanding of coverage bias in scWGS data (Zhang et al., 2015), Dong et al. reported a computational method that can correct amplification bias to reduce false positive SNPs resulting from PCR or sequencing errors (Dong et al., 2017). Although this new method still partially relies on GATK to identify new variants, it achieved better accuracy by removing false positive variants resulting from PCR error.

### Single cell transcriptome sequencing

Single cell RNA-seq data enables the discovery of exciting and new biological phenomena while presenting new challenges for data analysis. For example, single-cell RNA-seq can help us identify cell subtypes with unprecedented resolution, and reconstruct continuous cell lineages. Some early studies showed that identification of cell subtypes or reconstruction of cell lineage could be done manually by experts with sufficient biological prior knowledge using basic statistical methods (Xue et al., 2013; Treutlein et al., 2014). However, recently, huge datasets with extremely heterogeneous cell populations have precluded the feasibility of manual annotation, and many computational pipelines have been developed. For example, tools based on different theoretical frameworks have been developed to cluster cells based on their gene expression similarity, such as SINCERA (Guo et al., 2015), pcaReduce (Žurauskiene and Yau, 2016), SC3 (Kiselev et al., 2017), and SNN-Cliq (Xu and Su, 2015). Additional tools have been developed to reconstruct cell lineage by ordering cells according to computationally inferred pseudo-time (Trapnell et al., 2014; Cannoodt et al., 2016; Qiu et al., 2017). However, despite the availability of myriad computational software packages for clustering and lineage inference, few benchmarking studies have been done to compare their performance.

In addition to those two classical biological questions, the technical problem of imputation of missing values in single-cell RNA-seq data has recently attracted increasing attention. Single-cell RNA-seq, especially for cells captured by droplet-based methods, is often plagued by missing values due to drop-out events, leading to an exceedingly sparse depiction of the single cell transcriptome. Simply removing genes containing missing values restricts the analysis to only highly expressed genes. To overcome this problem, much effort has been made to impute missing values (Kiselev et al., 2017; Lin et al., 2017). These imputation methods can not only enable us to investigate lowly expressed genes but can also improve the performance of existing computational methods for other purposes by reducing noise from drop-out events.

### Single cell methylome analysis

Compared to bulk WGBS (whole genome bisulfite sequencing) data, the analysis of single cell WGBS requires distinct bioinformatics techniques due to the sparse and uneven coverage of scWGBS (single cell WGBS) libraries across the genome. Although many tools have been developed for bulk WGBS data analysis, these methods will fail if applied to scWGBS data directly. To make scGWBS data analysis possible, the first strategy is to merge data from single cells and analyze the merged data as a sample (Farlik et al., 2016). By combining data from many single cells (usually hundreds), the data coverage becomes high, and the bias from allele dropout is averaged out. However, this strategy cannot be used to address the heterogeneity of methylation among different single cells, because methylation data are merged and averaged among the cell population.

Aside from adapting scWGBS data to existing computational pipelines by merging data, the second strategy is to develop new methods specifically for scWGBS data, and many of these methods aim to aggregate methylation levels from adjacent CpG sites or regions with similar biological properties to overcome the sparseness of scWGBS data. For example, Smallwood et al. segment the genome into 5-kbp, non-overlapping bins and use average methylation level among bins as the feature for subsequent analysis (Smallwood et al., 2014). Similarly, by aggregating methylation signal on regulatory elements, we can reveal regulatory mechanisms behind the changes in the DNA methylome (Farlik et al., 2015). In these methods, each single cell is treated as a sample separately, thus enabling the discovery of DNA methylome heterogeneity among single cells.

Interestingly, besides aggregating existing methylation information to reduce noise, a method based on the deep neuronal network was recently developed, which infers missing methylation information from sequencing motifs (Angermueller et al., 2017). Although this method achieved high prediction accuracy for whole genome, its performance on low-methylated regions, the regulatory regions where methylation level influences gene expression greatly, were not satisfying. However, we believe that the prediction accuracy on LMRs can be further improved by incorporating more features into the same deep learning framework.

### Single cell sequencing for chromatin status analysis

Success in single cell genome and transcriptome sequencing inspired the development of single cell epigenome sequencing. So far, single cell ChIP-seq (Rotem et al., 2015) (Chromatin Immunoprecipitation Sequencing), DNase-seq, and ATAC-seq (Buenrostro et al., 2015) (Assay for Transposase-Accessible Chromatin using sequencing) has been reported from different groups. Since this type of single cell epigenome data has just begun to emerge, the related computational analysis methods are still in their infancy and only a few methods have been developed specifically for single cell data. For example, scChIP-seq and scATAC-seq have been developed to investigate histone modification and chromatin accessibility landscapes at single cell level (Buenrostro et al., 2015; Rotem et al., 2015; Corces et al., 2016), and the reads from one single cell are extremely sparse due to the low amount of DNA in a cell. To identify the regions that have histone modification or regions with open chromatin, reads from several dozen to hundred single cell libraries were pooled together, and only this “pooled library” has enough reads for conventional peak calling methods. In the subsequent analysis, these putative peaks will be used as guidance to aggregate sparse signal and remove background signal. Although this method enables the meaningful analysis of scChIP-seq and scATAC-seq without requirement of any new computational methods, concerns have been raised about the sensitivity of this strategy (Zamanighomi et al., 2017). Interestingly, methods designed for scATAC-seq analysis are emerging, such as chromVAR (Schep et al., 2017) and scABC (Zamanighomi et al., 2017). We believe these pipelines will also inspire the development of effective pipelines for scChIP-seq data.

## Application of single cell multi-omics methods

As described above, single cell multi-omics analysis integrates multiple data sets from the genome, epigenome, transcriptome, proteome, providing a unique chance to uncover novel biological processes. By extending and integrating methods developed for single-omics analysis, we can obtain a multi-channel molecular readout and utilize these features from multiple omics types to achieve a more comprehensive depiction of the state of a single cell. In combination with continuously advancing bioinformatic algorithms and computational resources, experimental collection of multi-omics data has allowed us to uncover increasingly important and complex insights.

The first application of single cell multi-omics methods is to identify cell subtypes from a heterogeneous cell population. Previously, for example, single cell RNA-seq approaches were shown to be effective in identifying cell subtypes such as human blood dendritic cells, monocytes, and neurons in human brain cortex (MacOsko et al., 2015; Ofengeim et al., 2017; Villani et al., 2017). Recently, single cell DNA methylation sequencing was also applied to study human brain cortex. By examining non-CpG methylation among single cells, they identified novel cell subtypes that were masked in scRNA-seq analysis (Luo et al., 2017). Epigenetic modifications such as DNA methylation are developmentally regulated and cell type-specific, yet stable over the life span, and therefore profiling the epigenome and transcriptome simultaneously can compensate for the limitation of single cell RNA-seq, which mainly yields information about highly expressed transcripts. Thus, different omics measurements can provide non-redundant information about cell identity and enable more detailed and more accurate dissection of complicated tissues.

Second, single cell multi-omics can be used to reconstruct cell lineage trajectories. Understanding cell lineage trajectories during the complete time course of multicellular animal development is the holy grail of developmental biology. DNA mutations, as well as epigenetic modifications gained during the cell division and passed to the daughter cells, can be used for lineage tracing, while the transcriptome of the matching single cells can reveal the concomitant alteration of gene expression and transcriptional cell fate change during cell proliferation and differentiaion. For example, cancer cells have extremely unstable genomes, and understanding cancer genome evolution is crucial for revealing “driver” mutations or copy number changes that cause carcinogenesis. Single cell multi-omics can not only help us determine the occurrence order of different mutations during cancer evolution, but can also reveal their functional consequences, such as alteration in gene expression, which will eventually help us identify the causal mutations that induce the transition from normal cell to cancer cell.

Lastly but most importantly, single cell multi-omics data provides the resolution to definitively reveal the relationship between different omics readouts. Correlation analysis between different omics is a prevailing approach to generate regulatory hypotheses between two omics data types. For example, cytosine methylation is among the best-studied epigenetic modifications and has been shown to regulate many critical biological processes. With both DNA and RNA sequencing data, DR-seq and G&T-seq have allowed us the ability to reveal correlation between copy number variation and gene expression level at a single cell scale. Further, scTrio-seq showed that large-scale CNVs caused proportional changes in RNA expression of genes within the gained or lost genomic regions, whereas these CNVs generally do not affect DNA methylation in these regions. Our work using scMT-seq not only showed allele-specific expression patterns based on SNV information, but also showed correlation of DNA methylation with allele-specific expression, providing new insight into the study of imprinting and its underlying mechanism. In the near future, multi-omics methods may be helpful for understanding the correlation between DNA mutations with epigenetic modifications and their effects on gene expression to reveal the mechanisms underlying interesting biological questions such as dosage compensation and X-inactivation, among others (Livernois et al., 2012; Graves, 2016). Inevitably, even with single cell multi-omics technology, we are still limited to identifying correlation but not causality. We therefore believe that single cell multi-omics, once combined together with experimental perturbation, will be effective in allowing us to understand causal relationships among omics data types.

Essential to all these applications is the development of computational approaches that help to integrate multiple data layers and to recover information lost due to the sequencing of minute amounts of biological material. Bioinformatic and computational techniques have advanced single cell multi-omics technology in several arenas, such as (1) imputation of “dropped-out” single cell measurements, (2) indirect measurement of another omics layer from a measured one (Farlik et al., 2015; Bock et al., 2016), and (3) mathematical and statistical quantification of multi-dimensional associations (Lane et al., 2017). Imputation methods pull information from groups of similar cells to help to restore measurements for molecules originally in very low abundance, such as lowly expressed RNA transcripts, filling in sparse data matrices for better representations of the original relationships (Van Dijk et al., 2017; Li and Li, 2018). Furthermore, as our knowledge of biological regulatory relationships increases, one data type may be able to serve as proxy for inference of another omics layer. For example, transcription factor binding or copy number alterations have been indirectly inferred from single cell methylation data (Farlik et al., 2015; Hou et al., 2016). Likewise, copy number information can be inferred from the single cell transcriptome (Tirosh et al., 2016), and chromatin state from the methylome (Guo et al., 2017). In addition, as single cell multi-omics technology becomes progressively high throughput, computational resources and time needed for processing of the raw data will be an important aspect in the flexibility of data analysis. Pipelines and new algorithms that streamline and shorten the computational time needed for data processing will be important for increasingly complex, multi-dimensional experiments. Raw files for each omic type must be separately processed, aligned, filtered, and quality-controlled in a manner that accounts for complications inherent in single cell measurements, such as low signal-to-noise ratio, technical amplification artifacts, and technical variation (Bock et al., 2016). Each omics layer of processed data is then assigned back to the single cell and co-analyzed with both mathematical and statistical models to reveal patterns of regulation. These new computational methods, while still nascent, allow us the capacity to bypass experimental limitations and expose excitingly novel relationships.

## Conclusions and future directions

Single cell multi-omics methods have provided countless opportunities to systematically understand biological diversity, and to identify rare cell types and their characteristics with unprecedented accuracy through integration of information from multiple omics levels, including DNA, RNA, and protein. These single cell multi-omics methods will play an important role in many diverse fields, and their applications are rapidly expanding, including (1) delineating cellular diversity, (2) lineage tracing, (3) identifying new cell types, and (4) deciphering the regulatory mechanisms between omics. Although some of the applications have been reported in initial studies, there are still many avenues open for exploration, and the further development of new multi-omics methods will also facilitate their increasing utility. It is anticipated that better performance of multi-omics methods will be generated based on the optimization of current single cell sequencing methods. There are currently several main challenges and thus opportunities for further development of single cell multi-omics technology: (1) Overcoming the limitations of current single cell sequencing methods will facilitate the development of more types of omics measurements on single cells. For example, outside of single cell DNA methylome analysis, there are other single cell epigenome sequencing methods such as scAba-seq (DNA hydroxymethylation) (Mooijman et al., 2016), single cell ATAC-seq (open chromatin) (Buenrostro et al., 2015), single cell Hi-C (chromatin conformation) (Nagano et al., 2013), and single cell ChIP-seq (histone modifications) (Rotem et al., 2015). However, due to limitations such as low genome coverage and high noise signals derived from locus dropout and PCR amplification, no reliable multi-omics approach based on these methods has been reported yet. Optimization of the existing single cell sequencing methods as well as newly developed methods will provide more opportunities to integrate diverse methods with transcriptomic analysis to reveal the relationship between epigenetic states and RNA transcription variation. (2) New approaches to isolate and label multiple types of molecules of the same single cell will help to increase the number of omics profiled in parallel, from dual-omics to triple-omics or more. Even multiple functional parameters of single cells could be included, such as with the development of patch-seq, which combined whole-cell electrophysiological patch-clamp recordings, single-cell RNA-sequencing, and morphological characterization to identify new cell types in the nervous system (Cadwell et al., 2016, 2017). (3) In contrast to the rich resources of experimental protocols, computational methods for single cell multi-omics data analysis have just started to emerge. New computational approaches tailored to the analysis of single cell multi-omics data will also substantially facilitate the application of the methods (Yan et al., 2017). In summary, with further development of multi-omics methods, the future will witness an even wider application of single cell multi-omics technology that will result in meaningful findings never before achieved.

## Author contributions

YH and YG: Conceived the structure of the manuscript; YH, YG, and QA: Wrote the manuscript; QA, BT, KS, and SF: Read and edited the manuscript.

### Conflict of interest statement

The authors declare that the research was conducted in the absence of any commercial or financial relationships that could be construed as a potential conflict of interest.
